# Efficacy of Coblation Technology in Treating Cervical Discogenic Upper Back Pain

**DOI:** 10.1097/MD.0000000000000858

**Published:** 2015-05-22

**Authors:** Liangliang He, Yuanzhang Tang, Xiuliang Li, Na Li, Jiaxiang Ni, Liangliang He

**Affiliations:** From the Department of Pain Management, Xuanwu Hospital, Capital Medical University, Xicheng Zone, Beijing, China (LH, YT, XL, JN, LH); Department of Anesthesia and Pain Management, Daqing Oifield General Hospital, No.9 Zhongkang Street, Saertu District, Daqing, Heilongjiang, China (NL).

## Abstract

Upper back pain originating from the cervical disk itself is defined as cervical discogenic upper back pain. Coblation procedures can provide therapeutic effects for neck and radicular pain related to contained cervical disk herniation. However, no studies have reported the performance of coblation procedures, particularly for treating cervical discogenic upper back pain. The purpose of this study was to evaluate the efficacy of coblation procedures in treating cervical discogenic upper back pain.

In a prospective, clinical, observational study, 28 consecutive patients with discogenic upper back pain underwent coblation procedures on the cervical disk with a percutaneous anterior approach. Pain visual analogue scale (VAS) scores, patient responses stating significant (≥50%) pain relief, significant (≥50%) reduction in pain medicine intake and Modified MacNab criteria were adopted to evaluate the pain intensity, degree of pain relief, and functional status after 12 months of follow-up.

The preoperative pain VAS score was 6.5 ± 1.1 (95% confidence interval [CI] 6.085–6.915), and the pain VAS score significantly decreased to 2.4 ± 1.3 (95% CI 1.929–2.928), 2.5 ± 1.5 (95% CI 1.963–3.109), 2.7 ± 1.4 (95% CI 2.157–3.271), 3.1 ± 1.6 (95% CI 2.457–3.686), and 3.1 ± 1.6 (95% CI 2.471–3.743) at 1 week and 1, 3, 6, and 12 months postoperatively, respectively (*P* < 0.05). Twenty-two (78.6%), 21 (75.0%), 20 (71.4%), 19 (67.9%), and 18 (64.3%) of the patients expressed significant pain relief at 1 week and 1, 3, 6, and 12 months postoperatively, respectively. 24 (85.7%), 23 (82.1%), 23 (82.1%), and 22 (78.6%) reported significant reduction in pain medication intake at 1, 3, 6, and 12 months postoperatively, respectively. According to the Modified MacNab criteria, the numbers of patients with “excellent” or “good” ratings were 22 (78.6%), 21 (75.0%), 20 (71.4%), and 18 (64.3%) at 1, 3, 6, and 12 months postoperatively, respectively. No serious complications were observed.

The findings of this study showed that coblation is an effective, safe, minimally invasive, and less uncomfortable procedure for the treatment of discogenic upper back pain.

## INTRODUCTION

Pain induced by stimulation of the disk itself is defined as discogenic pain, which shares clinical symptoms with radicular pain in most degenerated disk-related types of pain.^1,2^ Cervical radicular pain is characterized by a sharp lancinating pain, which often extends below the elbow into the forearm and hand, and the pathological mechanism is attributed to direct mechanical compression and inflammatory stimulation of the nerve root. However, cervical discogenic pain is described as a deep, dull ache that seldom spreads beyond the elbow.^1–3^ The pathological mechanism involves nociceptive fibers that penetrate into the inner annulus and the nucleus pulposus, along with vascularized granulation of tissue when annular ruptures occur in the inner and/or outer annulus, and ingrown nociceptors are irritated by inflammatory mediators and high intradiscal pressure.^4–8^

In studies of cervical discogenic pain distribution, upper back pain was reported when a stimulus was applied to the cervical disk.^3,9–11^ In 1943, Wedell and Feinstein performed electromyographic investigations and found that the painful area in the scapula was associated with cervical disk lesions; they concluded that local muscle spasms of the scapula were not induced by the painful nerve root but rather by a secondary source of pain.^3^ Later, the cervical intervertebral disk itself, and not the cervical or thoracic nerve root, was identified as the origin of the upper back pain through cervical discography,^2,3,5,9,10,12^ and this was defined as cervical discogenic upper back pain.

Currently, clinical studies have shown that the coblation procedure is effective for the treatment of cervical radicular/discogenic pain due to the removal of nuclear volume, reduction of intradiscal pressure, alteration of inflammatory agent expression, and interruption of nociceptive nerve endings.^13–20^ However, no studies have reported on coblation procedures that were performed to treat cervical discogenic upper back pain. In accordance with the pathological mechanism of cervical discogenic upper back pain and the therapeutic mechanism of the coblation procedure, we hypothesized that the coblation procedure is an effective method for managing cervical discogenic pain.

## METHODS

### Patients

After obtaining approval from the institution's Ethics Examining Committee of Human Research (Xuanwu Hospital, Capital Medical University, Beijing, China) and written informed patient consent, 28 patients who complained of cervical discogenic upper back pain related to contained disk herniation were scheduled to undergo the coblation procedure between September 2013 and January 2014 at Xuanwu Hospital.

The initial inclusion criteria for the coblation procedures were as follows: (1) unilateral cervical discogenic upper back pain without radicular pain and no neurological deficits, such as sensory or motor deficits or loss of reflexes; (2) a pain visual analogue scale (VAS) score ≥4; (3) a duration of pain ≥3 months; (4) restricted active and passive mobility of the cervical spine as observed by physical examination; (5) a contained herniated disk ≤3 mm not compromising more than 1/3 of the central spinal canal according to cervical magnetic resonance imaging (MRI); (6) an abnormal nucleogram with annular disruption in C4/5 and/or C5/6; (7) only grade 1 or 2 disk generation according to the Pfirrmann grading system; and (8) no bulge or protrusion observed by thoracic MRI.

Subsequently, stepper diagnostic and therapeutic procedures were performed to pursue the source of pain, including (1) trigger point injection to test for upper back myofascial pain syndrome; (2) scapulothoracic bursa injection to test for scapulothoracic bursitis; (3) thoracic medial branch block to test for thoracic facet joint syndrome; (4) thoracic epidural injection to test for thoracic radiculitis or discogenic pain; (5) cervical medial branch block to test for cervical facet joint syndrome; and (6) cervical epidural injection to test for cervical radiculitis or discogenic pain. If there were no responses to procedures (1) to (5) and there was a short-term response to procedure (6), the origin of the upper back pain was preliminarily considered to be the cervical disk.

Lastly, all the patients underwent cervical discography under mild sedation with 2 to 5 mg of midazolam, and a positive discography fulfilled the following criteria: reproducible VAS pain ≥7/10, <25 psi intradiscal pressure, <2.0 mL total volume, and 1 control disk according to the guidelines of the International Association for the Study of Pain. An injection was terminated if pain was produced, if a firm end point was reached, or if the disk was loose and accepting more medium than expected.^10^

Patients affected by coagulopathy, uncontrolled psychological disorders, disk herniation with sequestration, infection, spinal instability, spinal fractures, tumor, advanced spondylosis resulting in osseous foraminal stenosis, or disk space collapse as well as those with previous spinal surgery at the same level were excluded from the study.

### Coblation Procedure

The procedure was performed in an operating room using sterile techniques. The patient was placed in the supine position on the operating table, and a 10-cm cushion was placed under the shoulder to keep the neck slightly hyperextended. The patient received vital sign monitoring and oxygen supplied at 3 L per minute via a facial mask throughout the procedure. Before the procedure, an intravenous injection of etimicin (1.0 g) was administered as a prophylactic antibiotic. Patients received an intravenous injection of fentanyl (50 μg), and they were able to respond if a nerve root was irritated by thermal or mechanical stimulation. All the procedures were performed under local anesthesia.

**FIGURE 1:**
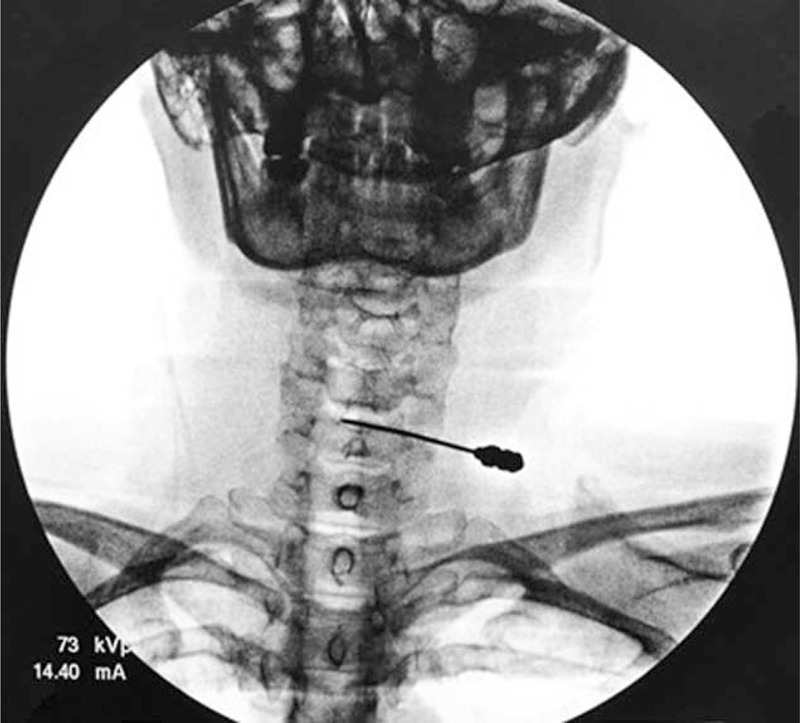
The introducer needle was advanced to the posterior annulus/nucleus junction at C5–6 as seen in the AP view. AP = anterior–posterior.

**FIGURE 2:**
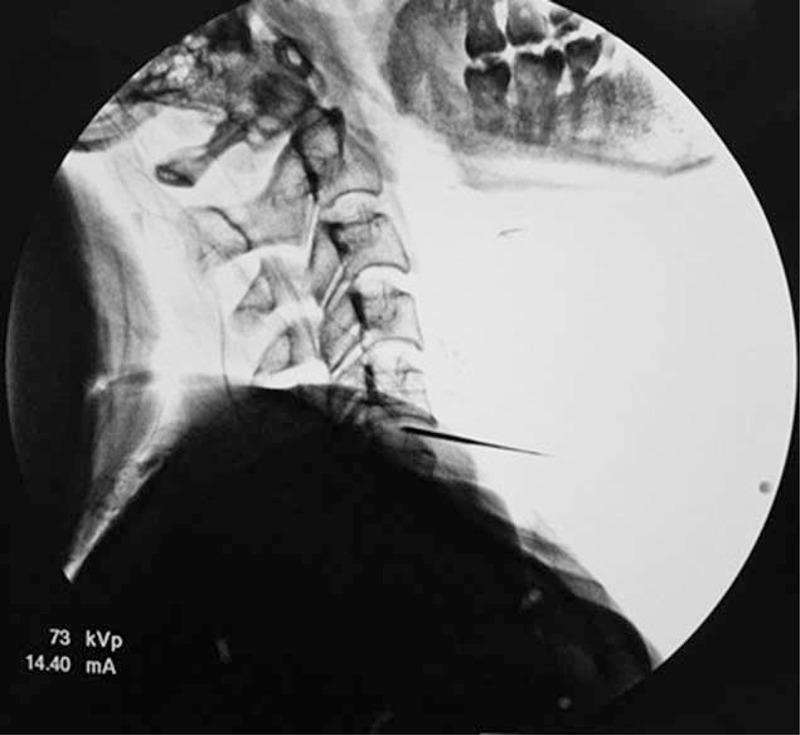
The introducer needle was advanced to the posterior annulus/nucleus junction at C5–6 as seen in the lateral view.

**FIGURE 3:**
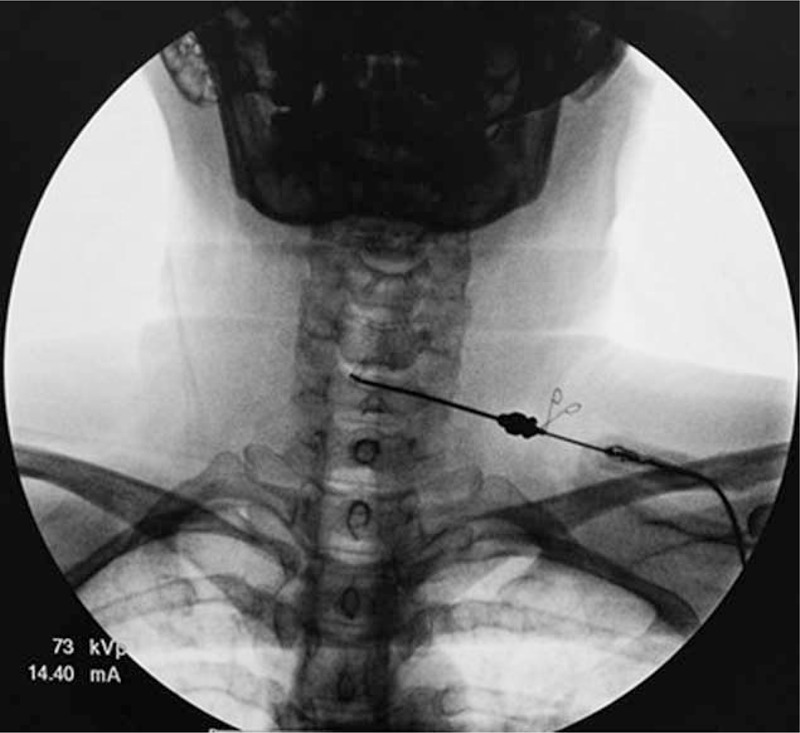
The tip of the coblation wand in the posterior annulus at C5–6 as seen in the AP view. AP = anterior–posterior.

**FIGURE 4:**
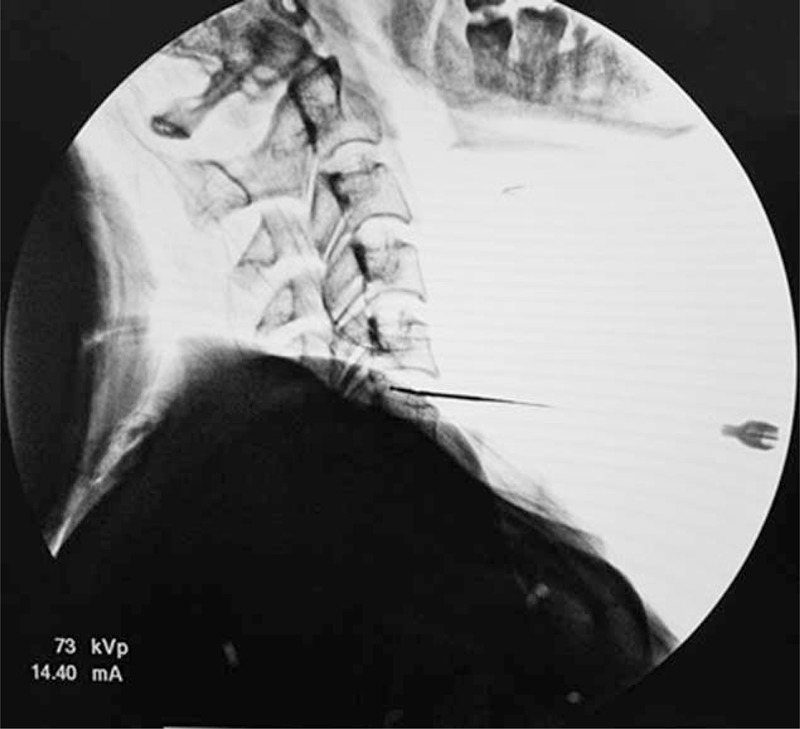
The tip of the coblation wand in the posterior annulus at C5–6 as seen in the lateral view.

First, the puncture angle was confirmed under fluoroscopic guidance with anterior–posterior (AP) and lateral views. Second, an 18-gauge, 8-cm introducer needle was advanced via a left or right anterior approach to the target disk. During the puncture process, the introducer needle was inserted slowly, and the advancement was immediately stopped when movement or paresthesia occurred in the patient's upper limb. Once the introducer needle entered the cervical disk, the needle was slowly advanced until the tip reached the opposite posterior annulus/nucleus junction, and the position of the tip was carefully checked in the AP (Figure 1) and lateral (Figure 2) views. Third, the coblation wand (UNITEC, China America United Technology [Beijing] Co. Ltd, China) was inserted into the introducer needle until the tip extended approximately 5 mm beyond the tip of the needle to ensure that the active portion of the wand was deployed into the annulus,^14^ and the position of the wand tip was again verified in the AP (Figure 3) and lateral (Figure 4) views. Fourth, coagulation was tested with a radio-frequency controller set at 2′ for 1/2–1 second to verify that there was no movement or paresthesia in the patient's upper limbs. Fifth, in ablation mode, the wand was rotated 360° with the radio-frequency controller set at an intensity of 2′ to ablate the disk material. Each ablation cycle was less than 10 seconds, and 3 ablation cycles were performed. Then, coagulation mode was conducted for 1 to 2 seconds with the controller set at an intensity of 2′ to denature adjacent materials and seal the channel. The tip of the introducer needle was then retracted approximately 5 mm to ensure that the active portion of the wand was deployed into the posterior third of the nucleus,^15^ and the ablation and coagulation were performed again following the above steps if no movement or paresthesia in the patient's upper extremities was reported. After withdrawal of the wand, 2 mL of 0.5% lidocaine was injected into the introducer needle tract. All the patients were subjected to bed rest in the supine position for 48 hours. After discharge from the hospital, the patients were advised to avoid strenuous activities. All the procedures were performed by 1 surgeon who has over 5 years of experience in performing coblation technology in the cervical disk, which is beneficial for avoiding clinical outcome bias based on surgical technique.

### Therapeutic Efficacy Assessment

Clinical improvement of pain after the coblation procedure was assessed by a pain VAS score recorded preoperatively and at 1 week and 1, 3, 6, and 12 months postoperatively. Significant pain relief (postoperative pain relief ≥50% compared with the preoperative state) was recorded at 1 week and 1, 3, 6, and 12 months postoperatively. The patient's functional status was evaluated as “excellent,” “good,” “fair,” or “poor” according to the Modified MacNab criteria and recorded at 1, 3, 6, and 12 months postoperatively. A significant reduction (≥50%) in pain medicine intake was recorded at 1, 3, 6, and 12 months postoperatively. Complications, such as hemorrhages, paresthesia, and infection, were recorded.

## STATISTICAL ANALYSIS

The patients’ demographic and baseline clinical data were analyzed descriptively. Repeated measures analysis of variance (ANOVA, a parametric test) was used to compare the improvement in pain VAS scores between the preoperative and postoperative time points. The Wilcoxon signed rank test was used to evaluate the extent of significant pain relief, the significant reduction in pain medication intake, and the functional status of patients after 12 months of follow-up. A value of *P* < 0.05 was considered statistically significant in all the analyses. Statistical analyses were performed using GraphPad Prism version 5.0 (GraphPad Software Inc., San Diego, CA).

## RESULTS

Twenty-eight patients suffering from discogenic upper back pain underwent the coblation procedure (15 males and 13 females). The mean pain VAS score was 6.5 ± 1.1 (range 5–9), the mean age was 48 ± 7 years (range 35–58 years), and the average duration of pain was 4 ± 2 years (range 1–8 years) (Table 1).

**TABLE 1:**
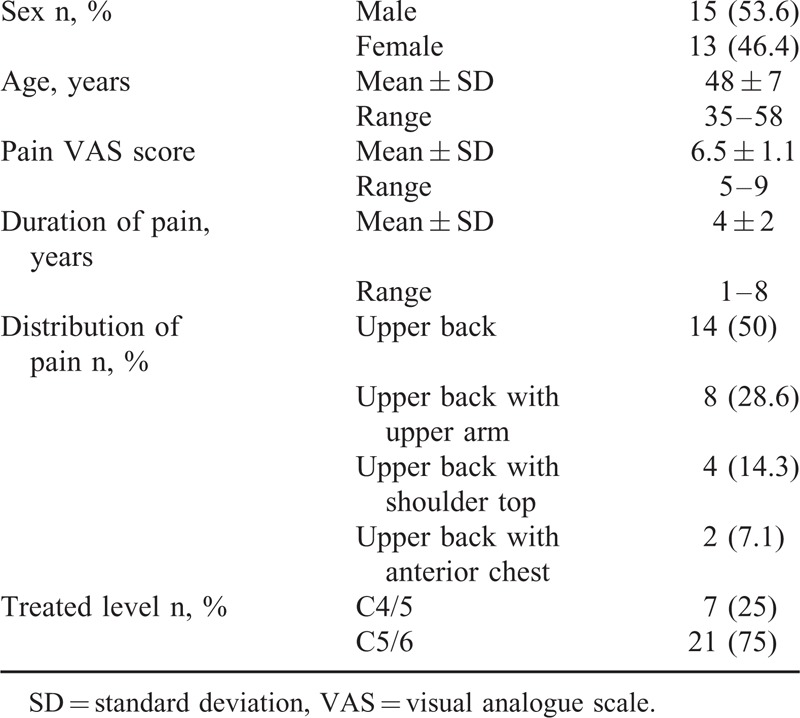
Demographic Characteristic

Before the operation, 14 patients complained of upper back pain (mainly located around the region of the vertebral border of the scapula and/or the superior and inferior periscapular regions); 8 patients complained of upper back pain with upper arm pain; 4 patients complained of upper back pain with upper shoulder pain (along the anterior border of the trapezius muscle); and 2 patients complained of upper back pain with anterior chest pain. Coblation was used to treat the C4–5 disk in 7 cases (37.5%) and the C5–6 disk in 21 cases (62.5%) (Table 1).

**FIGURE 5:**
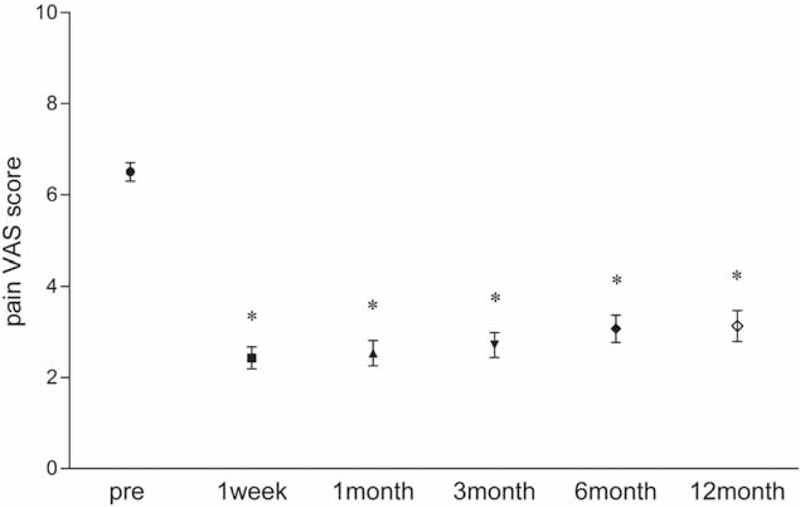
The pain VAS score preoperatively and at 1 week and 1, 3, 6, and 12 months postoperatively. The data are presented as the mean (error bars: 95% CI for the mean). ^∗^ Indicates a significant difference compared with the preoperative value. CI = confidence interval, VAS = visual analogue scale.

**FIGURE 6:**
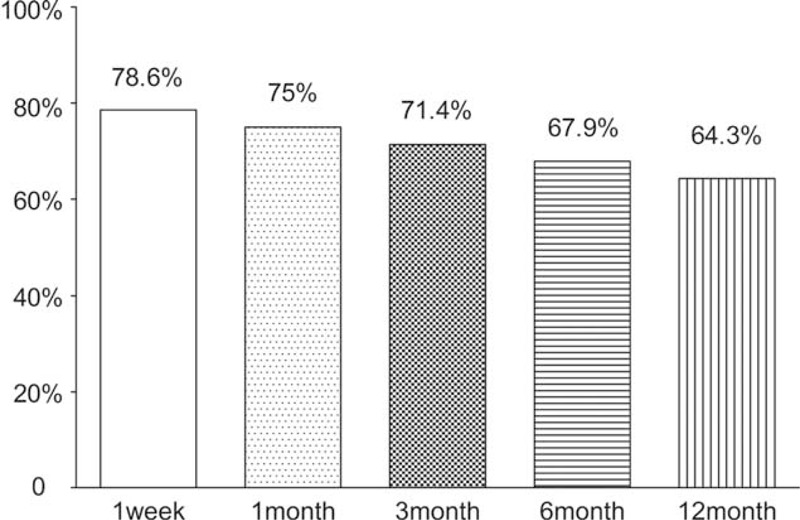
The proportion of patients reporting significant (≥50%) pain relief at 1 week and 1, 3, 6, and 12 months postoperatively.

Compared with the preoperative condition, the pain VAS score was obviously decreased at 1 week and 1, 3, 6, and 12 months postoperatively. The preoperative pain VAS score was 6.5 ± 1.1 (95% confidence interval [CI] 6.085–6.915), and the pain VAS score decreased to 2.4 ± 1.3 (95% CI 1.929–2.928, *P* < 0.05), 2.5 ± 1.5 (95% CI 1.963–3.109, *P* < 0.05), 2.7 ± 1.4 (95% CI 2.157–3.271, *P* < 0.05), 3.1 ± 1.6 (95% CI 2.457–3.686, *P* < 0.05), and 3.1 ± 1.6 (95% CI 2.471–3.743, *P* < 0.05) at 1 week and 1, 3, 6, and 12 months postoperatively, respectively (Figure 5). Only 2 patients reported that the pain VAS score decreased to 0 after 12 months of follow-up, but 22 (78.6%), 21 (75.0%), 20 (71.4%), 19 (67.9%), and 18 (64.3%) patients acquired significant pain relief at 1 week and 1, 3, 6, and 12 months postoperatively, respectively (Figure 6). At 12 months postoperatively, 4 patients did not experience significant pain relief; specifically, 2 patients complained of upper back pain (mainly located around the region of the vertebral border of the scapula and the superior and inferior periscapular region), 1 patient complained of upper back pain with pain in the upper shoulder, and 1 patient complained of upper back pain with anterior chest pain.

**FIGURE 7:**
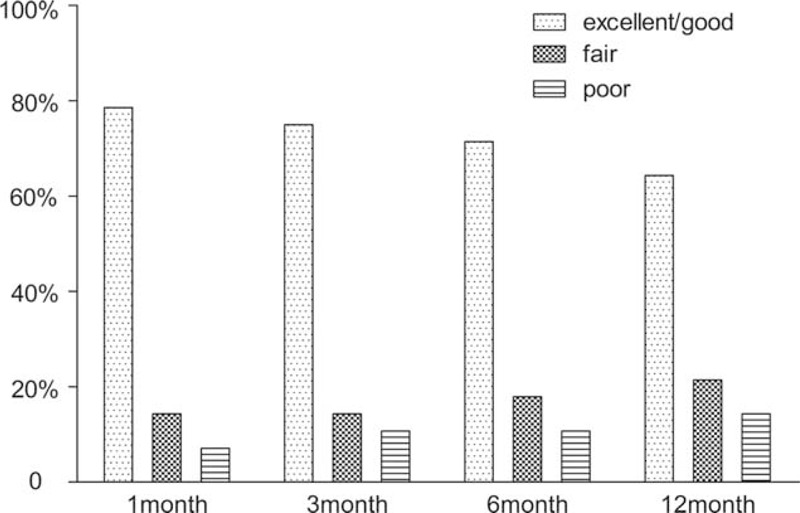
The proportion of patients who reported “excellent” or “good,” “fair,” and “poor” outcomes at 1, 3, 6, and 12 months postoperatively.

According to the Modified MacNab criteria, there were no differences in the proportion of patients with “excellent” or “good” ratings. At 1, 3, 6, and 12 months postoperatively, the respective numbers of patients with “excellent” or “good” ratings were 22 (78.6%), 21 (75.0%), 20 (71.4%), and 18 (64.3%); the respective numbers of patients with “fair” ratings were 4 (14.3%), 4 (14.%), 5 (17.9%), and 6 (21.4%); and the respective numbers of patients with “poor” ratings were 2 (7.1%), 3 (10.7%), 3 (10.7%), and 4 (14.3%) (Figure 7). Twenty-two (78.6%) patients needed to take analgesics preoperatively, and 7 (25.0%) patients needed to take analgesics to alleviate pain after 12 months of postoperative follow-up.

At 1, 3, 6, and 12 months postoperatively, the respective numbers of patient who reported a significant reduction in pain medication were 24 (85.7%), 23 (82.1%), 23 (82.1%), and 22 (78.6%). Thirteen (46.4%) patients reported soreness, and 3 (10.7%) patients experienced ecchymoma at the needle insertion site, but the symptoms completely disappeared within 2 weeks after the operation. No hemorrhages, paresthesias, or infections were observed.

## DISCUSSION

In this study, the coblation procedure significantly alleviated cervical discogenic upper back pain and improved the quality of daily life. The pain VAS score decreased from a preoperative score of 6.5 to a postoperative score of 3.1 at 12 months. At the end of 12 months of follow-up, the proportion of patients with significant pain relief was 64.3%, and the proportion of patients who reported “excellent” or “good” pain relief was also 64.3%. The efficacy of coblation in relieving cervical discogenic upper back pain for 12 months was demonstrated in this study.

Although the skin area corresponding to pain along the vertebral border of the scapula is supplied by the 2nd to the 7th thoracic nerve roots, and the muscles beneath these areas are supplied by the lower cervical nerve roots of the brachial plexus, a series of studies has confirmed that the cervical disk itself can provoke upper back pain.^2,3,9–12^ In 1996, Schellhas et al^9^ examined the cervical discogenic pain distribution from C3–4 to C6–7 in a small prospective study of 10 symptomatic and 10 asymptomatic subjects and found that upper back pain was provoked at C3–4 to C6–7 through discography. Four years later, Grubb and Kelly^10^ analyzed retrospective data from a 12-year experience using cervical discography from C2–3 to C7-T1 in 173 patients and found that C4–5 to C6–7 were responsible for the upper back pain. One year later, Slipman et al^11^ conducted a prospective multicenter descriptive study of pain provocation in 41 subjects undergoing cervical discography from C3–4 to C7-T1 and showed that interscapular pain can be produced from C3–4 to C7-T1.

However, to date, no diagnostic standard for cervical discogenic upper back pain has been published. Because upper back pain can potentially originate from thoracic radiculitis or discogenic pain,^21^ thoracic facet joint syndrome,^22^ scapulothoracic bursitis,^23^ cervical radiculitis or discogenic pain,^24^ cervical facet joint syndrome,^24^ or myofascial pain,^25,26^ it was difficult to enroll patients with this type of pain in this study. Therefore, to avoid the potential risk of misdiagnosis, all the subjects in this study received thoracic and cervical MRI and a stepper diagnostic and therapeutic procedure. Although MRI is considered the optimal imaging test to evaluate suspected disk degeneration due to the distinct image of the annular tear, significant disk annular tears often escape MRI detection.^27^ Therefore, all the patients underwent cervical discography to further localize the pain-generating site, and only patients who reported 70% provocation of concordant pain during discography were enrolled in this study. For all the enrolled subjects, restricted active or passive mobility of the cervical spine as observed by physical examination was useful diagnostic evidence for distinguishing upper back pain originating from the cervical disk rather than the thoracic disk.

In addition, the cervical discogenic pain distribution map plays an important role in diagnosing this type of pain. In 1959, Cloward^3^ first showed that pain occurs along the midline of the back or vertebral border of the scapula when a stimulus is applied to the midline or anterolateral surface of the disk, and the pain spreads out in a fan-shaped pattern over the scapula and into the upper arm during stimulation of the posterolateral surface of the disk. Consistent with the cervical discogenic pain distribution found in Cloward's study,^3^ this patients in this study suffered upper back pain mainly around the region of the vertebral border of the scapula and/or the superior and inferior periscapular region. Among these patients, only upper back pain was present in 14 patients, upper back pain with upper arm pain was present in 8 patients, back pain with upper shoulder pain was present in 4 patients, and back pain with anterior chest pain was present in 2 patients; these results were also similar to those from studies on provocative cervical discogenic symptom mapping.^9–11^

In this study, the C4–5 and C5–6 disks were selected as the targets, which coincided with the pain patterns described by Grubb and Kelly.^10^ In their study of cervical discogenic pain, similar pain distributions (including upper back pain) were produced at C4–5 and C5–6 by discography.

Coblation, as a minimally invasive surgery, has been recommended as an intermediate option between conservative and open surgical treatment for patients with degenerative disk and disk protrusion due to its ability to remove nucleus material, decrease the nucleus volume, reduce intradiscal pressure, and modify the intradiscal biochemical state.^28–30^ Although the safety and efficacy of coblation in treating radicular/discogenic pain related to contained disk herniation has been demonstrated by a series of clinical and experimental studies,^13–20,29^ the treatment of pain in the upper back or scapular region has rarely been studied. In Bonaldi's^20^ study, scapular pain, which is 1 symptom of radicular pain, was described in the inclusion criteria. In his study, 80% and 85% of patients reported “excellent” or “good” ratings of the clinical outcome at 2 and 6 months postoperatively, respectively. Compared with this study, our success rate was lower. However, different inclusion criteria (radicular pain vs discogenic pain) and surgical techniques may partly explain the different clinical outcomes.

Although positive clinical outcomes were demonstrated in this study, alternative diagnostic and therapeutic options should be considered before performing coblation therapy on the cervical disk, especially for upper back myofascial pain syndrome and thoracic facet joint syndrome. Because upper back pain more frequently results from myofascitis,^25,26^ upper back myofascial pain syndrome was first excluded by trigger point injection in this study. Additionally, up to 34% to 40% of chronic mid-back and upper back pain is caused by thoracic facet joint syndrome; therefore, it is necessary to rule out this type of pain through thoracic medial branch block, which is supported by reasonable evidence in managing chronic mid-back and upper back pain.^22^ A definite diagnosis of cervical discogenic upper back pain should be confirmed through strict differential diagnosis.

In our study, 22 (78.6%), 21 (75.0%), 20 (71.4%), and 18 (64.3%) patients expressed “excellent” or “good” clinical outcome ratings according to the Modified MacNab criteria at 1, 3, 6, and 12 months postoperatively, respectively, indicating a significant improvement in quality of life. However, 2 (7.1%), 3 (10.7%), 3 (10.7%), and 4 (14.3%) patients expressed “poor” clinical outcome ratings at 1, 3, 6, and 12 months postoperatively, respectively, and the cause was partly attributed to upper back pain, which may have resulted from multilevel degenerated disks causing cervical discogenic pain.^9–11^

In this study, 13 (46.4%) patients experienced soreness, and 3 (10.7%) patients experienced ecchymoma at the needle insertion site, which has been reported as the most common side effect from coblation procedures; however, the symptoms disappeared within 2 weeks after the operation.^31^ No complications, such as hemorrhages, paresthesias, or infections, were observed in this study. Although 1 case of infectious discitis was reported in Bonaldi's^20^ study, the symptoms of general infection were resolved by 8 weeks using a standard antibiotic regimen and rigid collar therapy. Therefore, the coblation procedure was demonstrated by this study to be a safe, minimally invasive, and less uncomfortable procedure for managing cervical discogenic pain.

A limitation of this study is the lack of either a historic or placebo control group. Conducting a blind, randomized, placebo-controlled study may be prohibitively expensive and logistically difficult in a clinical setting. Additionally, the sample size was small, and the results may not be generalizable to all patient populations. Nevertheless, this study does provide a preliminary framework for the planning of future prospective, randomized, controlled studies comparing coblation technology with conservative therapy in the treatment of discogenic upper back pain.

## CONCLUSION

In conclusion, dramatic improvements in the pain VAS score and the Modified MacNab criteria were obtained by coblation procedures for the treatment of cervical discogenic upper back pain. This method is an effective, safe, minimally invasive, and less uncomfortable procedure.
